# Steps towards standardized quantification of adult neurogenesis

**DOI:** 10.1038/s41467-020-18046-y

**Published:** 2020-08-26

**Authors:** Xinyu Zhao, Henriette van Praag

**Affiliations:** 1 Waisman Center and University of Wisconsin-Madison, Madison, WI 53705 USA; 2 Department of Neuroscience, School of Medicine and Public Health, University of Wisconsin-Madison, Madison, WI 53705 USA; 3 Brain Institute and Charles E. Schmidt College of Medicine, Florida Atlantic University, Jupiter, FL 33458 USA

**Keywords:** Cellular neuroscience, Neural stem cells

## Abstract

New neurons are generated in adult mammals. Adult hippocampal neurogenesis is considered to play an important role in cognition and mental health. The number and properties of newly born neurons are regulatable by a broad range of physiological and pathological conditions. To begin to understand the underlying cellular mechanisms and functional relevance of adult neurogenesis, many studies rely on quantification of adult-born neurons. However, lack of standardized methods to quantify new neurons is impeding research reproducibility across laboratories. Here, we review the importance of stereology, and propose why and how it should be applied to the study of adult neurogenesis.

## Abstract (web summary)

Adult neurogenesis is involved in several physiological and pathological processes, however standardization for the quantification of new born neurons is lacking. Here, the authors provide guidance to improve reproducibility and rigour in cell quantification.

## Introduction

The adult mammalian brain is hardwired for information processing while retaining a measure of plasticity that allows for adaptation and self-repair. Neurochemistry, physiology, and cell morphology can change in response to extrinsic and intrinsic stimuli^1^. However, the most striking form of adult structural plasticity is the addition of new neurons in discrete brain regions, such as the dentate gyrus (DG) of the hippocampus and subventricular zone (SVZ) of the lateral ventricles^2,3^. Neural stem cells in these brain regions can give rise to new neurons in adult mammals, albeit that the extent and duration of human neurogenesis remains unresolved^3,4^. Here, we will mainly discuss neurogenesis in the DG of the hippocampus, a brain area important for learning and memory^5,6^. However, the considerations for quantitative analysis apply to both neurogenic regions and extend to other cells and tissues.

## Adult neurogenesis

Adult-born hippocampal neurons mature over weeks of sequential changes in morphology, physiology and stage-specific expression of molecular markers^2,7^. In particular, radial glial cells, called type 1 cells, are neural stem cells that give rise to rapidly amplifying progenitors (type 2 cells) expressing proliferation markers (e.g. Ki-67, MCM2), that can develop into neuroblasts (type 3 cells), positive for doublecortin (DCX), PSA-NCAM, Calretinin (reviewed by refs. ^2,8^). These cells are gradually incorporated into the hippocampal network. One-week-old neuroblasts receive basal forebrain cholinergic as well as intrahippocampal excitatory glutamatergic and GABAergic inputs^9,10^. GABAergic neurotransmission becomes inhibitory at 2–3 weeks of new neuron age, concomitant with the growth of dendritic spines (reviewed by refs. ^2,11^). Around ~4 weeks of age the primary dendrite has extended multiple branches into the molecular layer, mature neuronal markers (NeuN, Calbindin) are expressed, axons have reached area CA3, and the cells receive afferent entorhinal cortex input. Adult-born neurons exhibit enhanced synaptic and structural plasticity for weeks to months during their integration into neural circuits^2,11^.

The functional relevance of new hippocampal neurons is based on regulatability by a variety of stimuli, including both behavioral and biological conditions^7,12^. Running has a strong positive effect on adult neurogenesis^13,14^ in association with enhanced synaptic plasticity and memory function (see recent reviews refs. ^12,15^). Exercise is now considered one of the most effective interventions for delay or prevention of aging-related neurodegeneration^15–17^. As such, enhancing adult neurogenesis may have therapeutic translational potential. However, the extent and amount of neurogenesis in humans remains unclear. The first evidence for human neurogenesis, obtained from post-mortem brain tissue from cancer patients treated with the thymidine analog bromodeoxyuridine (BrdU)^18^, was further supported by research correlating atmospheric ^14^C released by nuclear bomb testing with the incorporation of ^14^C into genomic DNA of dividing cells^19^. Histological staining using endogenous markers for cell cycle (e.g., Ki67, MCM2) and immature neurons (e.g., PSA-NCAM, DCX)^3,4,20,21^ provided corroborating evidence. In addition, three recent reports indicate there are large numbers of DCX^+^ cells in adulthood^22–24^, which are only reduced in Alzheimer’s Disease (AD) patients^23,24^. In contrast, three other recent studies question adult human hippocampal neurogenesis^25–27^. Analyses from gestation to old age show no new hippocampal neurons after the teenage years^25^, or even sooner, from early childhood^26,27^. Stereology was applied in studies reporting either the absence^26,27^ or presence^22–24^ of hippocampal neurogenesis. Differences in tissue fixation [formalin versus paraformaldehyde (PFA)], post-mortem interval (PMI), and staining protocols^23^, may contribute to discrepant results. Short PMI and the use of 4% PFA are considered optimal for labile antigens such as PSA-NCAM and DCX^23,28^. However, questions have been raised whether DCX is expressed by cell types other than immature neurons^21,29^, may be re-expressed in de-differentiating mature neurons^30^, or represent developmentally generated immature neurons^31^. Novel approaches^32^ may be needed to resolve the issue of adult human neurogenesis.

**Fig. 1:**
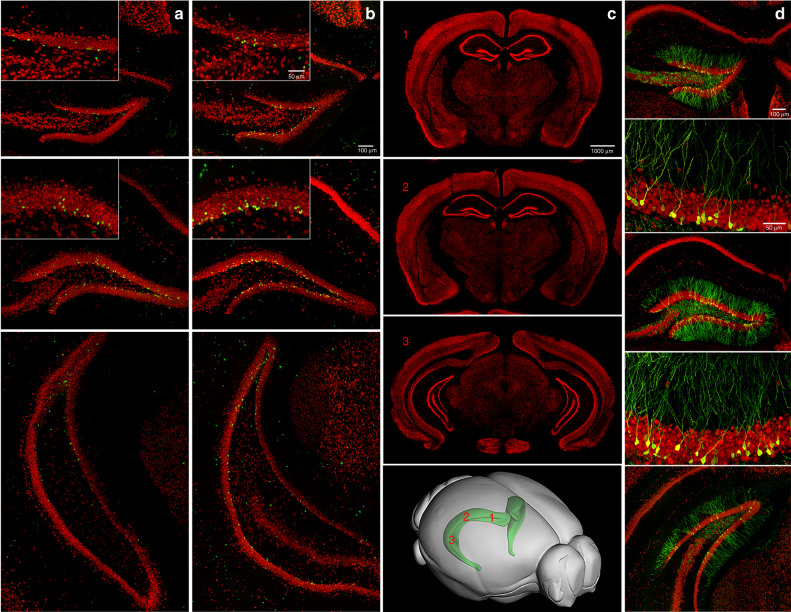
Adult neurogenesis in the dentate gyrus of the hippocampus. **a**, **b** Photomicrographs of brain sections throughout the rostral-caudal extent of the hippocampal dentate gyrus (DG), immunofluorescently double-labeled with BrdU (green) and NeuN (red), derived from adult C57Bl/6 mice housed under (**a**) control or (**b**) running conditions for 4 weeks. BrdU (50 mg/kg, i.p.) was injected daily for the first 10 days. The BrdU^+^ cell numbers in the DG granule cell layer are (**a**) 22, 46, 41, and (**b**) 44, 120, 52, respectively, from top to bottom. **c** Representative overview panels (distance from Bregma^95^ rostral to caudal: 1: −1.34 mm, 2: −1.94 mm, 3: −3.64 mm) of coronal sections through the hippocampus, labeled with NeuN (red). Lower panel: 3-D brain diagram shows the hippocampus (green) with numbers (1,2,3, red) corresponding approximately to section bregma levels in the panels above. **d** Photomicrographs of adult-born DG neurons with elaborate dendritic processes, targeted by retrovirus expressing green fluorescent protein (GFP) at one month after stereotaxic injection of retrovirus into the DG. Retrovirus labels dividing cells that develop into neurons over weeks. Coronal sections (40 μm), derived from adult C57Bl/6 mice, were co-labeled with NeuN (red).

While the direct translational link remains elusive, we consider interventions in rodents that promote neurogenesis, enhance cognition, and/or reduce stress, as important indicators of factors that can benefit human brain health. In rodents, adult cell genesis is readily detected and quantified using endogenous markers for neural progenitors and immature neurons, as well as by systemic injections of low doses of thymidine analogs, including BrdU, ethinyl- (EdU), chloro- (CldU) and iodo- (IdU) deoxyuridine, to label dividing cells. Staining for these analogs in combination with neural markers can be used to quantify all stages of neurogenesis, from neural stem cell proliferation to differentiation into mature neurons. In addition, sequential administration of each analog can be used to study different stages of the neurogenic process in the same animal^33,34^. Of particular interest is EdU, which can be visualized without the use of tissue denaturing or antibodies^34^. Furthermore, in rodents intrahippocampal injection of retroviral vectors to label dividing progenitor cells allows for birth-dating and functional analyses^9,10,14,35–37^ (Fig. 1). Another effective approach is the use of transgenic animals in which a fluorescent reporter is driven by promoters specific for different stages of new neuron development (e.g. Nestin, GFAP, SOX2, Tbr2, DCX, HopX) (for review see ref. ^38^). For instance, expression in type 1 cells can be driven by promoter sequences for *Nestin*^39,40^, Notch target gene *Hes5*^41^, lysophosphatidic acid receptor^42^, and selectively Lunatic fringe (Lfng), a regulator of Notch signaling^43^. Combining these models with thymidine analog labeling can provide quantitative developmental stage-specific data (for review see refs. ^2,38^).

## Rationale for standardized analysis of adult neurogenesis

The broad range of conditions that affect adult neurogenesis has resulted in intense interest in the field across disciplines. To determine a causal link between adult neurogenesis and behavior, researchers have used genetics, neuropharmacology and, x-irradiation as methods to modulate new neuron production and function^44^ followed by assessment of hippocampus-dependent behavioral changes in wildtype rodents as well as in animal models of neurodevelopmental, neurodegenerative and neuropsychiatric conditions such as autism, epilepsy, dementia, depression, schizophrenia, and addiction^40,45,46^. As such the need of researchers to measure adult neurogenesis as a read-out of genetic or pharmacological manipulations, as well as to gain mechanistic insight into brain functions and behaviors has increased exponentially. While this speaks for the significance of the phenomenon, the field lacks the required analysis standardization to improve data reproducibility and comparison among studies and across laboratories. A more detailed and systematic quantification of adult neurogenesis could improve research in the adult neurogenesis field.

The main goal of this review is to help researchers to achieve rigorous and reproducible results. While the points raised are relevant for research on adult neurogenesis, they also apply more broadly for cell quantification in tissue. We provide a few examples of studies with different approaches to neuronal quantification to illustrate why methodological standardization would benefit the field. Indeed, many adult neurogenesis studies provide detailed information about cell counting procedures and stereological parameters used^40,47–49^. However, other publications present a less informative methods section, where the total number of new cells in the DG, the number of sections counted, or the distance between sections are not described^50,51^. These seemingly trivial details may have profound impact on data interpretation. An interesting example is the equivocal effect of memantine, an N-methyl-D-aspartic acid (NMDA) receptor antagonist used for the treatment of AD^52^, on adult neurogenesis. Several reports suggest that memantine strongly stimulates adult neurogenesis^53–56^. Conversely, other researchers reported no change in hippocampal BrdU^+^ cells at 28 days post-labeling, and no change in DCX^+^ cell number using the same drug dose^57^. When we take a close look at the quantitative methods for BrdU counting several differences are apparent. In the studies where a large memantine-induced increase was observed, cells were counted in thin 14 μm micron brain sections, in every 6th section throughout the rostral-caudal extent of the DG^53–56^. On the other hand, researchers reporting no change^57^ used 40 μm thick brain sections and counted BrdU^+^ cells using 1 in 12 sections spanning the rat DG. None of the studies used stereology and whether their sampling rates were sufficient is unknown. The effects of memantine on adult neurogenesis remain unclear. This example underscores the importance of establishing a uniform standard for adult neurogenesis analysis.

The adult neurogenesis field would greatly benefit from an effort across laboratories to standardize the protocols used to quantify the number of new neurons, regardless of the marker used. There are several aspects of the neurogenic process that make it challenging to quantify. In the adult neurogenic zone, proliferating cells are initially overproduced and only a limited percentage of the cells generated reaches maturity, resulting in a rapidly changing cell number during the first few weeks of their development in the adult brain^44^. Neurogenesis is strongly influenced by genetic background^58,59^, species^60^, and aging^61^. Furthermore, the regional distribution of newly born neurons is not uniform. The number of new cells is higher in the dorsal as compared to the ventral DG^62^. In addition, the neurogenic response to behavioral or pharmacological stimulation differs across the DG, consistent with functional differences between the dorsal (spatial navigation and memory) and ventral (mood and emotion) aspects of the structure^63,64^. Running increases adult neurogenesis in the dorsal DG^65,66^, whereas depression^67^ and antidepressants reportedly affect the ventral aspect^68,69^. As described in more detail in the next section experimenters should perform a comprehensive analysis of the entire extent of the DG of the hippocampus and ensure that the relevant cells are (co)-stained with markers indicative of cell division (e.g., BrdU, Ki67, MCM2, SOX2, PCNA, β-Tubulin) and/or neurogenesis (e.g., DCX, Prox-1, BrdU/Calbindin, BrdU/NeuN), (Fig. 1).

## Design-based stereology

**Fig. 2:**
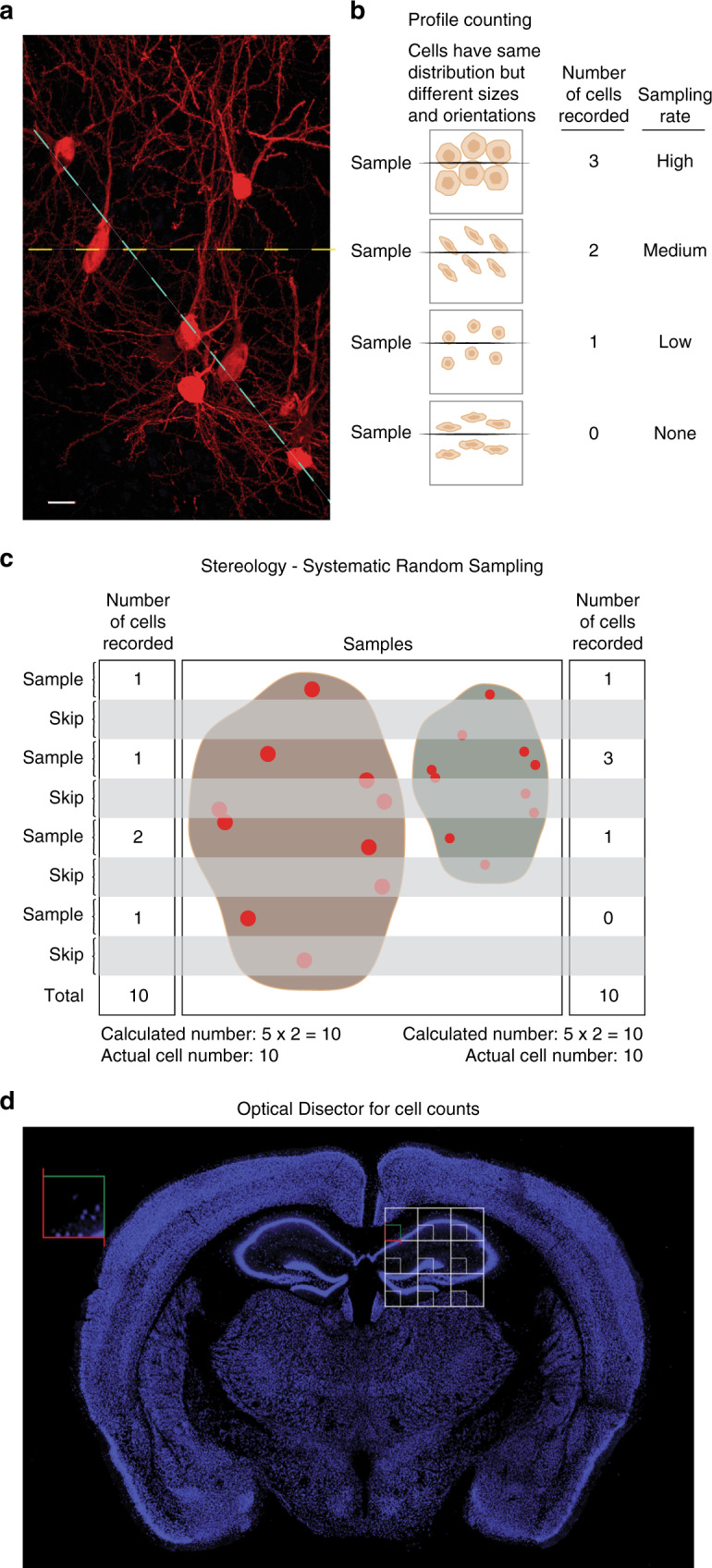
The benefit of using design-based stereology with random sampling to quantify cells. **a** Photomicrograph of entorhinal cortex neurons with variable shapes and uneven distribution. The image was derived from a C57Bl/6 mouse injected into the DG with rabies virus expressing mCherry, as a retrograde tracer^11^ (40 μm horizontal section). Scale bar, 10 μm. The dotted lines illustrate two different potential focal planes for microscopic visualization with different cell numbers. **b**. Schematic illustration showing that cell profile counting, using a ‘representative” thin section (dark line) of tissue, is heavily influenced by the size and orientation of the cells of interest. In a thin section, a larger cell (top sample) has more chances to be represented and therefore counted than a smaller cell (third sample from top). The orientation of cells that are not round can significantly change the outcome of the counting, as illustrated by the second sample (2 cells are counted) versus the fourth sample (no cell is counted). The results of profile counting may not reflect the actual cell number. **c** Schematic illustration showing that design-based stereology with serial randomized sampling throughout the entire three-dimensional tissue (example shows 1 in 2; ssf = 1/2) yields more accurate counts. The tissues on the left and right both have 10 cells. Despite the differences in size of the tissues and cells, the results are close to the actual cell numbers. **d** Cell quantification using the optical disector. A sampling grid (white) overlays the region of interest (ROI) for quantification, the hippocampus. The intersections of the grid within the ROI define the position of the counting frames (red and green) at which the sections are probed at high magnification. The cells within the volume of the tissue in each 3-D optical disector probe are counted if they do not touch any surface or the green surface, whereas those that touch red are not included. Photomicrograph of a 40 μm coronal section through the mouse brain labeled with NeuN (blue).

Quantitative analysis of cell numbers, cell density, and volume of brain regions has been an important part of neuroscience research since mid-1800. However, neuroscientists quickly realized that results obtained from observing a few three-dimensional brain sections were variable and inconsistent due to uneven distribution of cells, subtle changes not obvious to the eyes, as well as distortion and shrinkage resulting from tissue processing. Early on these issues led to contradictory conclusions that hampered advancement of the neuroscience field and other areas of research [reviewed by ref. ^70^]. For example, profile counting in which one representative thin section of tissue is selected for quantification of the number of cells within the region of interest, can lead to significantly different results depending on which tissue level representative sections are chosen from (Fig. 2a). In addition, the resulting cell numbers vary significantly depending on the size and shape of the cells and orientation of the section plane relative to the shape of the cells (Fig. 2b). Indeed, a larger cell has higher chances of being included in a thin representative section than a smaller cell, or as compared to a cell that has undergone shrinkage due to biological reasons or during histological processing. An elliptical cell is also more sensitive to the orientation of the representative thin section plane than a round cell. Profile counting has the most profound impact on the analysis of relatively rare cells, such as adult-born new neurons^4,20^.

Stereology was developed to systematically and reproducibly perform quantitative assessments of both subtle and large differences between control and experimental (or healthy and pathological) conditions^70^. The term stereology was first introduced by Hans Elias in early 1960s to define a method to correct observations made in two-dimensional quantification to infer three-dimensional information (e.g., cell number and region volume)^70^. These early stereology protocols were difficult to apply due to dependence on cell geometry and specific correction formulas. Design-based stereology was developed in early-1990s and has been successfully used in neuroscience research^71^. Design-based means that the protocols for analysis, including the nature of the probes used and the sampling schemes, are designed in such a manner that the experimenter does not need to consider the geometry (size, shape, spatial orientation, and spatial distribution) of the cells of interest, leading to more robust data^72^. This method was quickly adapted by neuroscientists for analysis of total cell number, cell distribution, regional volume, and fiber tracks in the nervous system. It also ensures rigorous quantitative analysis of the size, shape, and number of objects^71^, when properly used (Fig. 2c). For instance, this methodology provided evidence that the number of hippocampal neurons remains stable with aging, and that changes in synaptic density may mediate memory deficits^72,73^. For a comprehensive description of the use of Design-based stereology in neuroscience, see Schmitz and Hof^71^.

**Fig. 3:**
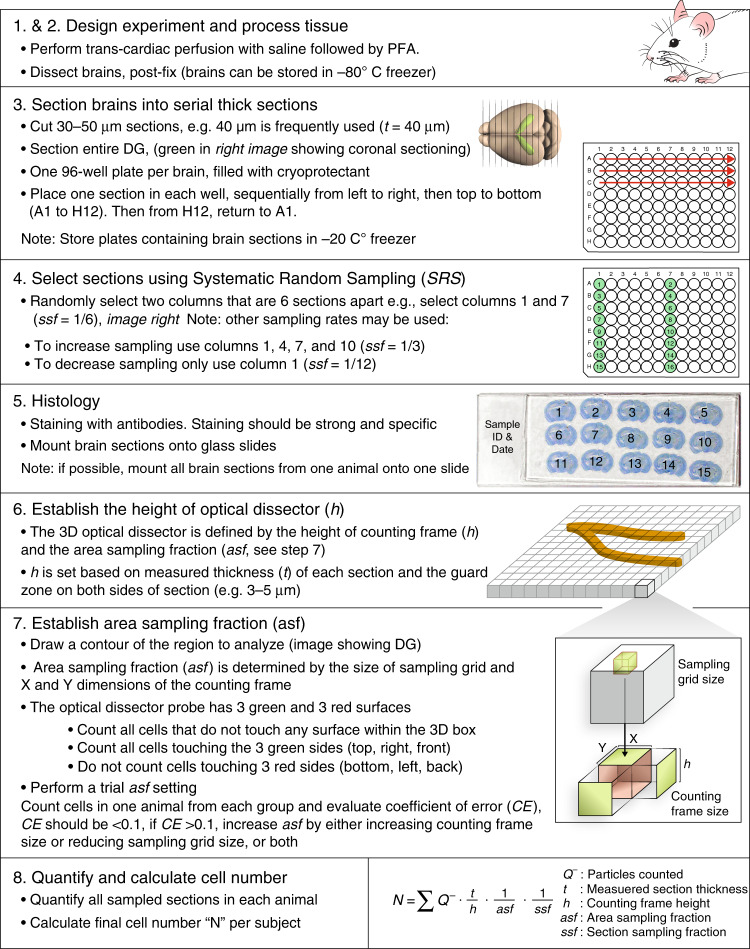
Design-based stereology work flow for quantitative analysis of adult neurogenesis. Optimal results of experiments can be obtained by following these steps: 1. Experimental design. 2. Tissue processing for optimal histological analysis. 3. Sectioning into thick (e.g., 40 μm) sequential sections and store in long-term tissue storage reagent (e.g. cryoprotectant). 4. Selection of representative sections using systematic random sampling (SRS). 5. Histological staining of the tissue with appropriate antibodies for cell types of interest. 6. Establishing the height of counting frame for optical fractionator (h). 7. Establishing the area sampling fraction (asf) to define the fraction of the specimen that will be quantified to represent the entire specimen. This is done by setting the sampling grid and defining the size of the counting frame (X and Y dimensions) for optical fractionator. 8. Quantification of sampled regions using optical fractionator and calculation of total numbers of markers in the specimen using the parameters set above and the number of cells counted.

Several key principles for Design-based stereology ensure accuracy of the data. These include: (1) systematic and random sampling (SRS), (2) calculation of total cell numbers instead of densities, (3) counting of cells, not cell profiles, (4) use of thick tissue sections to visualize cells throughout 3-D probes in a known tissue volume, and (5) sensitive and specific staining to clearly identify the cells of interest. Specifically, to achieve sound quantitative results, the brain sections selected for analysis and the microscopic fields chosen for counting must be representative of the entire brain region of interest. All parts of the structure must have an equal chance to contribute to the analysis by using SRS. For this purpose, thick serial sections are taken uniformly throughout the structure and then sampled at defined intervals (e.g. every 6th section) for quantification. After staining of the SRS sections, a grid is superimposed over the region of interest (ROI), for example, the hippocampus (Fig. 2d). The intersections of the grid within the ROI define the position of systematically and randomly selected microscopic fields (counting frames) at which the sections are probed at a higher magnification. The cells that become visible while focusing through the volume of the tissue within each 3-D probe are counted by clear criteria that define inclusion or exclusion of the cell. In particular, the optical disector probe has three red and three green surfaces. Cells that do not touch any surface and cells that touch green are included, whereas those that touch red are not. Together with guard zones at the top and bottom of the sampling probe, this ensures that no cell is counted twice (Fig. 3). These criteria seem simple but are not easy to meet, therefore, proper experimental planning and practices should be in place.

## Application of design-based stereology to the analysis of adult neurogenesis

Accurate quantitative analysis of adult neurogenesis requires a systematic approach: optimal experimental design, high quality and well-planned tissue collection and processing, careful considerations and good practices (Fig. 3). Here, we provide methodological details for the quantification of adult hippocampal neurogenesis.

1. Experimental Design. The animals used in the study should all be the same age (+ one week) as adult neurogenesis changes rapidly with aging^61,74^. If the researchers choose to use thymidine analogs then reagent preparation, injection frequency, and dosage must be carefully determined. For example, BrdU is recommended to be administered via intraperitoneal (i.p.) injection, at the same time each day. Repeated^74^ low daily doses (50 mg/kg) for 10 days, rather than a single high dose (e.g., 300 mg/kg)^75^ of BrdU injections is suggested to reduce toxicity or labeling of cells undergoing DNA repair^34,76^. In addition, not all thymidine analogs have the same solubility. For example, large amounts of BrdU may not dissolve into solution within an adequate injection volume, and reliance on a single injection may carry more risk of skewing results than repeated administration. Also, BrdU should be prepared fresh every day, filtered, and protected from light. Finally, the time interval between injections and brain tissue collection will depend on whether the researchers are studying cell genesis or survival.
2. Tissue collection and fixation. It is essential to properly perfuse the animal with physiological saline (room temperature) to clear the brain tissue of blood to prevent high background histological staining. Following saline, the animal is perfused with PFA (ice-cold) prepared in phosphate buffer, shortly before the procedure (<24 h) to minimize polymerization that can affect the quality of tissue fixation^77,78^. Although trans-cardiac perfusion is a standard procedure, performing it properly and consistently is not trivial. Rigorous training according to a well-established protocol should be required in each laboratory^78,79^. Without good tissue fixation, subsequent precise quantitative analysis will be much more difficult, which is particularly important for human studies which rely on immersion fixation^18,22–28^. Immersion fixation is used when transcardiac perfusion is not feasible, e.g. postmortem human brain tissue or tissue that will be in part used for biochemical or molecular assays (which needs to be flash frozen upon collection) and in part used for anatomical analysis (e.g., histology or immunohistochemistry). Following perfusion (or immersion fixation), post-fixation duration is generally 24 h after which the tissue is placed in 30% sucrose solution for cryoprotection. It should be considered, however, that certain antibodies may be particularly sensitive to post-fixation intervals^77^ and experiments using these reagents should be planned accordingly.
3. Tissue processing and storage. The next step is cutting brain tissues into sections with defined thickness. There are several key considerations in this step to meet the requirements of unbiased design-based stereology. Modern design-based stereology uses 3-dimensional (3D) stereological probes and optical disector method, which requires minimal shrinkage and the use of thick rather than thin sections. However, if the sections are too thick, antibody penetration becomes problematic, therefore, 40–50 μm brain sections are optimal. Sectioning can be performed using frozen tissue on a sliding microtome, or non-frozen material on a vibratome. Use of cryoprotectant [1 L PO4 (0.1 M) + 600 mL ethylene glycol + 500 mL glycerol]^58,80^ to fill tissue section collection plates ensures minimal changes in tissue shape and size, and allows for long-term storage of samples in a −20 °C freezer. Paraffin embedding of tissue can also be used, however, tissue deformation and shrinkage may occur^81^. It should be noted that good and consistent sectioning technique is important because uneven cutting surface and loss of cell nuclei may occur when the knife hits the tissue surface. Care should be taken to minimize these variations by controlling tissue temperature, and by using a high quality and sharp knife on a level stage. Setting a counting guard zone (see below) in the optical disector will further reduce the impact of sectioning imperfections.
  Design-based stereology requires that the experimenter has access to the entire region of interest, which is achieved by systematic random sampling of sections from exhaustive section series encompassing the entire region. Therefore, the part of brain containing the entire hippocampus must be completely sectioned and all sections must be collected. Again, this requires the experimenter to be precise and consistent. To aid random sampling of tissues, we use 96-well plates to store sequential serial sections.
4. Systematic random sampling (SRS). Selection of brain sections containing the hippocampus for histology and quantification. Design-based stereology requires SRS which is achieved at two levels: inter-section and intro-section (see the sixth step). At inter-section level, all sections containing the hippocampus must have equal probability to be selected. To achieve this, we randomly select 1 in 6 columns from the 96-well plates containing the sections (see the third step). The intra-section level of SRS is achieved through the selection of microscopic fields in a systematic random manner that guarantees that all parts of the DG have the same chance of contributing to the sampling (see the sixth step).
5. Histology. The histological markers used in staining brain sections and analysis should be carefully selected. The signals should be strong and specific and have been validated with the appropriate controls. For example, a negative control can be derived from mouse tissue in which the specific antigen is not present or in which a relevant gene is deleted, and a positive control could be use of a transfected cell line expressing the antigen for the primary antibody. Antibodies can be assessed by western blotting. A clean signal band at the correct molecular weight should be detected in tissues with, but not without, the antigen. However, western blot immunoreactivity does not necessarily predict immunoreactivity in fixed tissue sections^77^. The histological markers for adult neurogenesis are well-characterized^34^. For BrdU immunostaining, a widely used procedure is to perform antigen retrieval by incubation of tissue in formamide/2X saline-sodium citrate (2 h at 65° C), followed by incubation in 2 N HCl (30 min at 37° C) to denature the DNA (e.g., refs. ^48,49^). BrdU staining in rodent tissue can also be performed without the antigen retrieval step (e.g., ref. ^60^). To determine whether the BrdU-labeled progenitors have become immature (DCX) or mature neurons (NeuN, Calbindin), staining can be combined with labeling for these markers. After staining the experimenter must define the criteria for cell quantification. For example, either the top or the maximal perimeter of a BrdU^+^ nucleus or DCX^+^ cell body can be used. For a cytosolic marker such as DCX counting the cell body may yield more consistent results than quantifying dendritic processes. However, it is also important to consider that immature cells express DCX for several weeks while their morphology continues to evolve. Researchers have therefore made sub-categories of DCX^+^ cells based on morphological features^80^. Overall, it is important to select only one unique identifier for each cell marker for all study samples.
6. Establishing the height of optical disector (*h*). Quantification using the optical fractionator method requires a 3D counting probe called optical disector. The size of a 3D optical disector is determined by the size of the counting frame (X and Y dimensions) and the height of sample frame (Z dimension). To determine the appropriate height for optical disector (*h*), the experimenter must first measure the thickness of the brain sections because they change, in most cases shrink, as a result of tissue processing. For example, a frozen brain section cut at 40 μm may have thickness (“measured thickness” or *t*) at about 30 μm after staining and mounting. In addition, tissue sectioning may lead to loss of nuclei on the surface of the tissues leading to inaccurate counting, therefore the experimenter needs to set “guard zones” at both sides of the tissue to reduce the bias. For fluorescently stained brain sections with 30 um mounting thickness, we use 3–5 μm guard zone on each side. The height of optical disector should be less than the measured section thickness minus guard zones. The height of optical disector (*h*) over measured section thickness (*t*), or the ratio of h/t, so called the height of sampling fraction (*hsf*), is used for calculating final cell number.
7. Establishing area sampling fraction (*asf*). The most critical step in proper application of design-based stereology is determining the optimal fraction of the specimen to be analyzed in order to represent the entire specimen. The parameter to define this fraction is called the area sampling fraction (*asf*). The optical disector requires that a contour is drawn to include the region of interest on each tissue section at low magnification. The contour does not need to be exact but should include the entire DG area, as well as one cell body area at the inner surface of the SGZ to ensure the inclusion of newborn cells. Only cells within the contour (region of interest) are counted. Then a sampling grid is laid on top of the contour. The sampling grid determines the number of sites sampled within the contour. The denser the grid, the more sites are sampled. Finally, each sampling grid contains a counting frame that defines the x and y dimension of the 3D optical disector probe. The larger the x and y, the more tissue is sampled. Together, the density of sampling grid and size of counting frame determine the area sampling fraction (*asf*), which is the fraction of the region of interest that will be sampled. Sufficient sampling is necessary to provide an accurate estimate of the cell number in the entire brain region. Denser sampling grid and a bigger counting frame (bigger optical disector probe) means that more cells will be sampled, which may provide a more accurate estimate, but will require more time to complete the counts.
  The coefficient of error (CE) helps the experimenter to determine the optimal sampling method that yields an accurate estimate within the least amount of time^82^ Fortunately, there are a number of hardware and software systems (e.g., StereoInvestigator, MicrobrightField; SRC Biosciences) that can assist with this calculation. It is good practice to run pilot analyses with various *asf*, based on literature and prior experience, to assess CE which will help the experimenter to determine whether the sampling is sufficiently rigorous. For example, for quantification of new neurons in the DG of young adult C57Bl/6 mice, we use a previously set counting frame and grid to count about 150–300 cells/DG to optimize CE and adjust these parameters to CE < 0.1.
8. Quantification of optical fractionator parameters. The final step is calculation. Once you have counted the cells using the optical fractionator, the estimated total cell number in this brain region is calculated using the parameters you have set, including section sampling interval (*ssf*), area sampling fraction (*asf*), and height of sample fraction (*hsf*). (Total cell number = total cells counted × 1/*ssf*  × 1/*asf* × 1/*hsf*). The data are presented as total number of new cells per DG per subject. Because the above parameters are essential for obtaining rigorous quantitative results and for evaluating and comparing among different studies, they should be documented in the experimental record. The following details should be provided in the Methods of published manuscripts:

- Number, frequency and dose of BrdU or other thymidine analog injections: _________
- Section cut thickness: ____________
- Measured section thickness: ____________
- Section evaluation interval: ___________
- Rostral-caudal (dorsal-ventral) extent of sections taken: ___________
- DG volume / overall cell count for new cell density measures
- Guard zones: ____________
- Height of optical disector: ___________
- Size of counting frame dimensions: _________________
- Size of counting grid: ___________________

## Special considerations

For the purpose of quantitative analysis, we consider the optical disector method preferable. To count samples with very low cell numbers, the sampling grid and probe size can be adjusted to increase area sampling fraction (*asf*) to cover the majority of the DG in each section. Conversely, when the numbers of positive cells are high such as in certain transgenic mouse models^39,42^, *asf* is reduced within the allowance of coefficient of error. However, as the purchase of a stereology system can be costly, there are alternatives that could be utilized. One alternative approach is complete counting, which is either counting every cell in every single section or every cell in all SRS-selected sections. This could be performed by an experienced scorer^83^, particularly in cases where the number of newborn neurons is sparse, such as in aged animals^74^.

An alternative approach to the optical disector, to estimate neuron number in thin histological sections, is the physical disector^84^. This approach utilizes of a series of section pairs that are separated by a known distance. The section pairs consist of a reference section and a look-up section. Cell cross-sections that are seen in one section (reference) but not in the other (look-up) are counted as positive cells. The count of all of these positive cells is multiplied by the reciprocal of the fraction of the volume being sampled to obtain an unbiased estimate of total cell number. The physical disector has been utilized to quantify the number of immature DCX^+^ neurons in confocal images^85^. However, recently developed confocal stereology techniques^86^ make it even easier to use the optical disector method, setting counting frames (*asf*) and optical disector height (h), to generate a 3D optical disector. The Z-stack confocal images are collected at each sample site and then probed using the 3D optical disector. This method can generate cell counts of single or multiple markers, as well as cells co-labeled with multiple markers^73^.

Cell counts may be accompanied by volume analysis of the region of interest. The volume of the DG can be obtained using the area of the contour drawn for cell counting (total analyzed volume = area of contour x mounting thickness × 1/ssf). This volume can be used to calculate density of adult-born cells (= total cell number/total volume). However, although volume analysis indicates whether there is a change in DG size, it alone does not provide total granule cell number. For precise estimates of DG volume and total granule cell number, staining brain sections using a nuclear dye (e.g., Hoechst) followed by the optical fractionator for determining granule cell number and another stereology method designed for volume analysis, Cavalieri-point-counting^87^, can be used^58^. It should also be noted that certain conditions, such as epilepsy or stroke, may make accurate volume analysis difficult. Volume analysis can be important in transgenic mouse models that result in expansion or shrinkage of the brain. For example, two studies analyzed septal cholinergic cells in mutant mice for the low affinity nerve growth factor receptor p75. One group reported an increase in septal cell number based on counts of cells per section^88^. However, the opposite result was reported by another laboratory, after a detailed stereological analysis that accounted for the change in septal volume that occurred as a result of the mutation^89^.

Furthermore, as for any scientific investigation, appropriate statistical methods must be used for both experimental design and data interpretation to ensure rigor and reproducibility. For quantitative rodent adult neurogenesis studies, the sample size, depending on the design of the study and statistical power analyses, will typically be around 6–10 animals per group to allow for parametric statistics. A larger *n* per group will reduce potential variability resulting from individual differences between subjects, systemic BrdU injections, and tissue processing steps.

## Remaining challenges

To evaluate changes in adult neurogenesis, obtaining accurate cell counts is the first necessary step. Differing results between groups studying rodent or human adult neurogenesis may be resolved using standardized cell counting and optimal staining methods. In addition, the experimenter should be aware that cell number quantification is only the start of neurogenesis research. Analysis of expression of genes (markers) corresponding to neuronal maturation, dendritic and axonal extension, pathfinding, synaptogenesis, physiological changes, and integration are integral parts of adult neurogenesis^2,12^. To understand whether an increase or decrease in neurogenesis is functionally relevant and meaningful, physiological and fine morphological measurements, as well as connectivity of the cells with other brain areas should be evaluated^11,66^. In some cases, even though the number of new cells may not change, maturation and integration of adult new neurons may be altered^37,80^. Finally, given the role of adult neurogenesis in brain plasticity, behavioral outcomes should be measured as a potential consequence of changes in adult neurogenesis. A number of DG / adult neurogenesis-sensitive behavioral assays have been successfully used in this regard, including the Morris water maze, fear conditioning, and novel object location. In particular, adult hippocampal neurogenesis is considered important for pattern separation, the ability to distinguish between very similar events or stimuli. Several behavioral tests, including touchscreen, radial arm maze, and contextual fear conditioning paradigms have been developed to assay pattern separation in rodents [see for recent reviews refs. ^7,15,90^].

## Future directions

Adult neurogenesis is an exciting discovery that has captured extensive attention and imagination because it has reversed the long-held view that the adult mammalian brain is static and has provided tools and hope for potential treatment of difficult brain disorders. Since the first discovery of adult neurogenesis more than 50 years ago^91^, the field of adult neurogenesis research has evolved over time from a potential artifact of BrdU labeling (e.g. DNA repair) to a well-established phenomenon with translational relevance^61^. The adult neurogenesis field is now approaching its maturity and would greatly benefit from an effort across laboratories to standardize the protocols used to quantify the number of new neurons, regardless of the marker used. While this may not be straightforward due to differences among experimental design and readout, much can be achieved by following guidelines for quantitative analysis to ensure that every new neuron counts.

Finally, we want to point out that the concerns we raised pertaining to standardization of adult neurogenesis studies can be extrapolated to many other research areas dealing with similar issues. Beyond the application of standard stereological practices, we hope that the future will bring novel automated analytical methodologies and the development of automated 3D counting algorithms. Recent progress has been made in this regard with programs to quantify synapses^92,93^ and complete neural cell counts^94^ by machine learning. Such methods would further support rigor and reproducibility between investigators and benefit science in general, and so we hope, the next generation of neuroscientists in particular.
